# Chemical and mechanical properties of dual-polymerizing core build-up materials

**DOI:** 10.1007/s00784-022-04455-4

**Published:** 2022-03-28

**Authors:** Matthias Kelch, Bogna Stawarczyk, Felicitas Mayinger

**Affiliations:** Department of Prosthetic Dentistry, University Hospital, LMU Munich, Goethestrasse 70, 80336 Munich, Germany

**Keywords:** Core build-up materials, Martens hardness, Elastic indentation modulus, Biaxial flexural strength, Degree of conversion, Raman spectroscopy

## Abstract

**Objectives:**

To investigate the chemical (degree of conversion (DC)) and mechanical properties (Martens hardness (HM), elastic indentation modulus (E_IT_), and biaxial flexural strength (BFS)) of four dual-polymerizing resin composite core build-up materials after light- and self-polymerization.

**Materials and methods:**

Round specimens with a diameter of 12 mm and a thickness of 1.5 mm were manufactured from CLEARFIL DC CORE PLUS (CLE; Kuraray), core·X flow (COR; Dentsply Sirona), MultiCore Flow (MUL; Ivoclar Vivadent), and Rebilda DC (REB; VOCO) (*N* = 96, *n* = 24/material). Half of the specimens were light-polymerized (Elipar DeepCure-S, 3 M), while the other half cured by self-polymerization (*n* = 12/group). Immediately after fabrication, the DC, HM, E_IT_, and BFS were determined. Data was analyzed using Kolmogorov–Smirnov, Mann–Whitney *U*, and Kruskal–Wallis tests, Spearman’s correlation, and Weibull statistics (*p* < 0.05).

**Results:**

Light-polymerization either led to similar E_IT_ (MUL; *p* = 0.119) and BFS (MUL and REB; *p* = 0.094–0.326) values or higher DC, HM, E_IT_, and BFS results (all other groups; *p* < 0.001–0.009). When compared with the other materials, COR showed a high DC (*p* < 0.001) and HM (*p* < 0.001) after self-polymerization and the highest BFS (*p* = 0.020) and Weibull modulus after light-polymerization. Positive correlations between all four tested parameters (*R* = 0.527–0.963, *p* < 0.001) were found.

**Conclusions:**

For the tested resin composite core build-up materials, light-polymerization led to similar or superior values for the degree of conversion, Martens hardness, elastic indentation modulus, and biaxial flexural strength than observed after self-polymerization. Among the tested materials, COR should represent the resin composite core build-up material of choice due to its high chemical (degree of conversion) and mechanical (Martens hardness, elastic indentation modulus, and biaxial flexural strength) properties and its high reliability after light-polymerization. The examined chemical and mechanical properties showed a positive correlation.

**Clinical relevance:**

The chemical and mechanical performance of dual-polymerizing resin composite core build-up materials is significantly affected by the chosen polymerization mode.

## Introduction

A loss of natural tooth substance, that is caused by caries or trauma and frequently occurs in combination with an endodontic treatment, calls for a prosthetic restoration with single- or multi-unit fixed dental prostheses (FDPs). To imitate the natural tooth with its soft dentin core and hard enamel shell, the treatment of choice customarily consists of a resin composite core build-up and subsequent restoration with a FDP made from an alloy or ceramic. The estimated 5-year success rate of such “conventional” restorations is reported to be 94%, while endocrowns, where a preceding core build-up is foregone, show a success rate of 78% [1]. In the day-to-day practice, dentists frequently perform the necessary pretreatments (e.g., a root canal filling) and the subsequent core build-up in one sitting, while the final preparation, impression taking, and provisional FDP are tackled in the next appointment. With the core build-up being in extensive direct contact with oral structures in this time period, the chemical properties of the employed materials (e.g., their residual monomer content) are of paramount importance [2, 3]. Furthermore, the overall stability of the FDPs is influenced by the chemical and mechanical properties of the employed resin composites [4, 5]. The choice of core build-up material thus holds a profound impact on the longevity of the prosthetic restoration. Since their market launch, the properties of resin composites have been continuously improved by modifying their composition [6]. In general, resin composites are made up of an organic polymer matrix, (predominantly inorganic) fillers, coupling agents (that establish a chemical bond between organic and inorganic components), initiators, inhibitors, accelerators, and other additives. The introduction of dual-polymerizing resin composites, that contain both light- and self-polymerization initiators, expanded the indication spectrum of this material group. As a result, the adhesive luting of endodontic posts and subsequent core build-up can, as of today, be performed with only one material, thus shortening treatment times and reducing inherently weak material interfaces and processing errors [7]. Different resin composite core build-up materials vary in their composition and, in consequence, in their chemical and mechanical performance [5, 8–10]. Whereas a resin composite’s filler fraction has been reported to positively correlate with its mechanical properties [11, 12], fillers can also inhibit the polymerization reaction and thus impair the achieved degree of conversion (DC) [13, 14]. For a light-induced initiation of the polymerization reaction, this observation can be explained by a high filler content, a small filler size or a mismatched refractive index of fillers and polymer matrix increasing the reflection of light [15]. The formation of free radicals can also be impaired by pigments absorbing light and thus reducing the amount of light energy that reaches deeper layers [9, 16]. The chemical and mechanical properties of resin composite core build-up materials can furthermore be affected by the amount and distribution of photo- and chemical initiators, accelerators, inhibitors, and the chosen polymerization mode [8, 17–19]. While previous investigations reported a higher crosslink density [18] following light-polymerization, the influence of the polymerization mode on the chemical and mechanical properties of resin composite core build-up materials remains unclear [10, 17–20].

The chemical properties of resin composites are standardly evaluated by determining the DC, either by Fourier-transform infrared or Raman spectroscopy. The higher the DC, the lower are the amount of elutable substances and the residual monomer content [21]. From a clinical point of view, this parameter is pivotal, as it determines a material’s biocompatibility. A resin composite’s mechanical performance can be evaluated by measuring its Martens hardness (HM), elastic indentation modulus (E_IT_), and biaxial flexural strength (BFS). The HM and E_IT_ yield information about a material’s surface properties. HM has been reported to impact a material’s resistance to wear, with a low Martens hardness correlating with a low wear of the antagonist [22]. Although a core build-up material is only subject to wear during a limited period between doctor’s appointments, the determination of the HM is important as it also quantifies a resin composite’s resistance to deformation during loading. The measurement of the E_IT_, an analog to the Young’s modulus, allows an assessment of the elastic properties of a material. The higher the E_IT_, the stiffer is the material and the lower are its damping properties. This should be considered when evaluating a material’s behavior during function, as this property impacts the stress states between different restorative materials and influences the interactions in the masticatory system. The determination of the biaxial flexural strength of a geometric body allows an additional means to predict the performance of a dental restorative material during loading and enables an evaluation independent of the material’s surface properties.

The aim of this investigation was to investigate the chemical and mechanical properties of different core build-up materials after light- and self-polymerization. For this purpose, the DC, HM, E_IT_, and BFS of four core build-up materials were examined. The tested null hypotheses stated that the tested chemical and mechanical properties were neither affected by the polymerization mode nor the type of core build-up material used.

## Material and methods

**Fig. 1:**
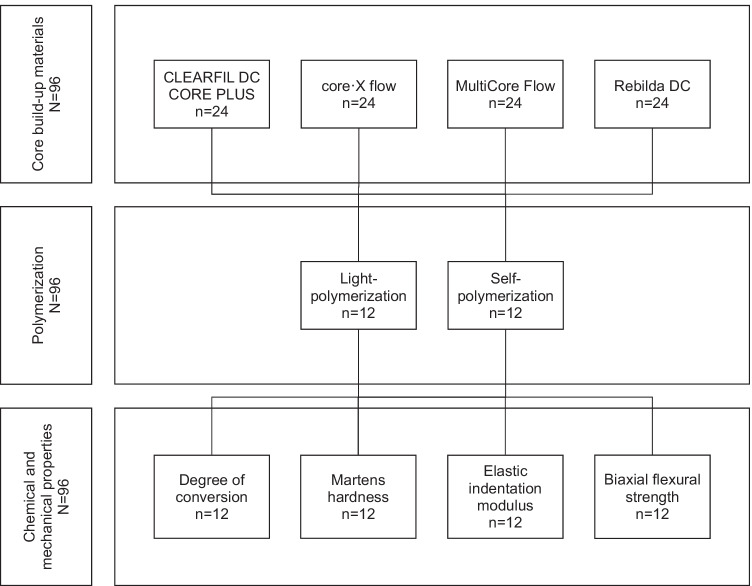
Study design

The DC, HM, E_IT_, and BFS of four different core build-up materials were investigated after light- and self-polymerization (Fig. 1, Table 1).

**Table 1:** Materials, abbreviations, manufacturers, lot numbers, processing guidelines, and composition used

Material	Abbreviation	Shade	Manufacturer	Lot no	Processing guidelines		Composition*
					Light-polymerization	Self-polymerization	
CLEARFIL DC CORE PLUS	CLE	White	Kuraray, Tokyo, Japan	2S0216	10 s	6 min	< 5% Bis-GMA, < 5% TEGDMA, hydrophilic aliphatic dimethacrylate, hydrophobic aromatic dimethacrylate, silanated barium glass filler, silanated colloidal silica, colloidal silica, dl-Camphorquinone, aluminum oxide filler, initiators, accelerators, pigments
Core·X flow	COR	Tooth-colored	Dentsply Sirona, Konstanz, Germany	200,400,083	20 s	2–3 min	2.5– < 10% Urethane modified Bis-GMA dimethacrylate resin, 2.5– < 10% UDMA, 2.5– < 10% TMPTMA, 2.5– < 10% TEGDMA
MultiCore Flow	MUL	White	Ivoclar Vivadent, Schaan, Liechtenstein	Z001M5	10 s	4–5 min	≥ 10– < 20% Ytterbium trifluoride, 10–25% Bis-GMA, ≥ 2,5– < 10% UDMA, ≥ 2,5– < 10% TEGDMA, ≥ 0,1– < 1% dibenzoyl peroxide
Rebilda DC	REB	White	VOCO, Cuxhaven, Germany	2,022,105	40 s	5 min	10– < 25% UDMA, 5–10% DDDMA, 2.5–5% Bis-GMA

^*^As provided by the manufacturers

*Bis-GMA* bisphenol A-glycidyl methacrylate, *DDDMA* Dodecanediol dimethacrylate, *TEGDMA* triethylene glycol dimethacrylate, *TMPTMA* propylidynetrimethyl trimethacrylate, *UDMA* urethane dimethacrylate

### Specimen preparation

A total of 96 specimens were manufactured at room temperature with a hollow acrylonitrile butadiene styrene mold (SD Mechatronik GmbH, Feldkirchen-Westerham, Germany) to form round disks with a diameter of 12 mm and a thickness of 1.5 mm. The mold was slightly isolated with petroleum jelly (Vaslinum, Fagron GmbH, Barsbüttel, Germany) prior to the injection of the core build-up material. Half of the specimens were light-polymerized (Elipar DeepCure-S, 3 M, Seefeld, Germany), while the other half cured by self-polymerization. For each core build-up material, the manufacturers’ processing guidelines listed in Table 1 were meticulously adhered to. The surface of each specimen was carefully wiped with 96% ethanol (Otto Fischar GmbH, Saarbrücken, Germany). The measurements of the DC, the HM and E_IT_, and BFS were performed directly after light- or self-polymerization in immediate succession.

### Measurement of the degree of conversion (DC)

The DC was examined with a Raman spectrophotometer (inVia Qontor, Renishaw, New Mills, UK) after a careful calibration of the system. To record the Raman scattering of the unpolymerized material (R_unpolymerized_), the unpolymerized core build-up materials were applied on a microscope slide. Per material group, twelve measurements were taken to calculate an average value for R_unpolymerized_. The Raman spectra of the unpolymerized and polymerized (R_cured_) specimens were recorded with a single mode laser operating with a wavelength of 785 nm. For each specimen, one Raman spectrum was measured at 100% laser power and an irradiation time of 10 s. The obtained data were processed with WiRE 4.4 (Renishaw) software by subtracting the baseline and determining the peaks at 1610 cm^−1^ and 1640 cm^−1^ using the curve fit function. The DC was calculated with the following equation:

$$DC (\%)=100*[1-\frac{{R}_{cured}}{{R}_{unpolymerized}}],\mathrm{ where\,\, }R = \frac{{band\,height\,at\,1640\,cm}^{-1}}{{band\,height\,at\,1610\,cm}^{-1}}$$

**Fig. 2:**
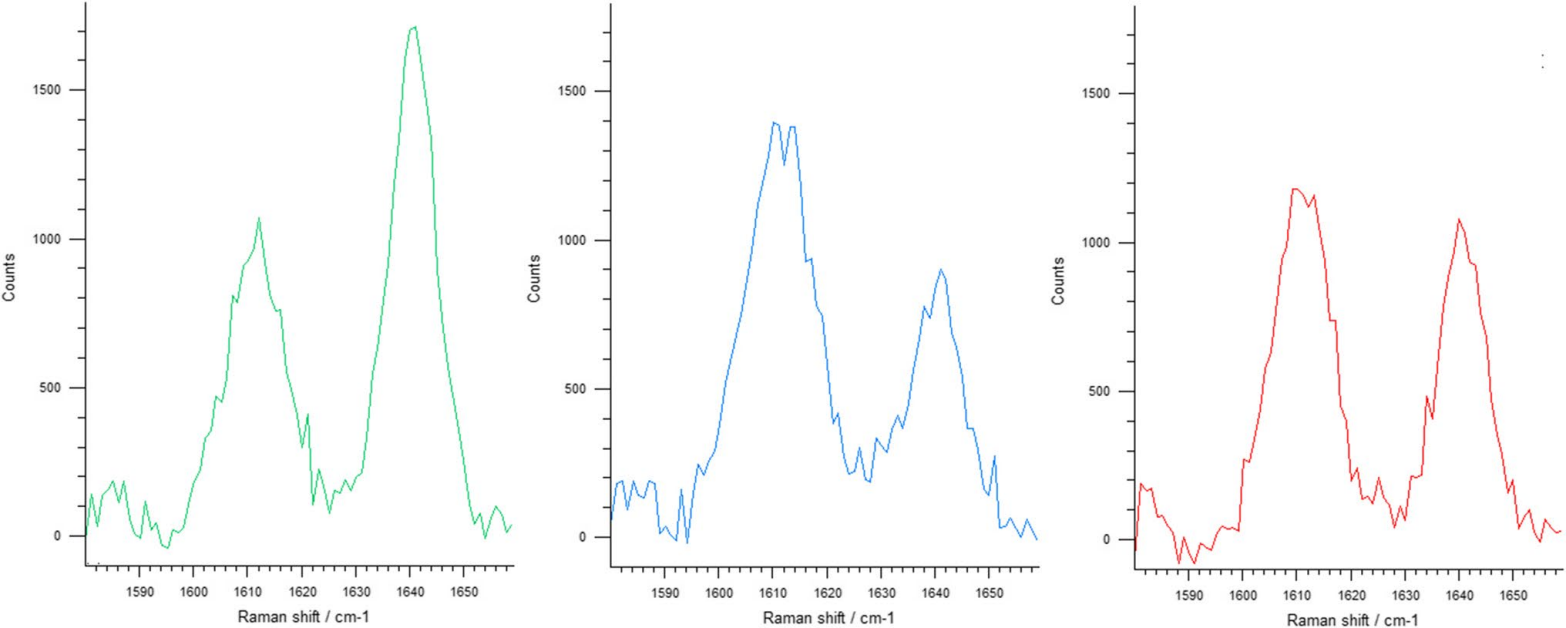
Raman spectra for the different polymerization modes: unpolymerized (left), light-polymerized (center), and self-polymerized (right) for a CLEARFIL DC CORE PLUS specimen

The Raman spectra for the different polymerization modes are presented in Fig. 2.

### Measurement of the Martens hardness (HM) and elastic indentation modulus (EIT)

The HM and E_IT_ were determined using the testing machine ZHU 0.2 (ZwickRoell, Ulm, Germany). The mounted diamond indenter in the form of a Vickers pyramid was vertically pressed into the specimens’ surface. Specimens were loaded with 9.8 N for 2 s at three different positions. HM an E_IT_ values were computed using TestXpert v.12.3 Master (ZwickRoell) with the equations given in ISO 14577–1 [23]:

$$HM=\frac{F}{{A}_{S}\left(h\right)}$$

where *HM* is the Martens hardness, *F* is the test force [N], *A*_*S*_* (h)* is the area of the indenter penetrating the surface at distance h from the tip [mm^2^] and

$${E}_{IT}=(1-{v}_{S}^{2}){\left(\frac{\sqrt[2]{{A}_{p}\left({h}_{c}\right)}}{\sqrt{\pi S}}- \frac{(1-{v}_{i}^{2})}{{E}_{i}}\right)}^{-1}$$

where *E*_*IT*_ is the elastic modulus of the indenter [N/mm^2^], *A*_*p*_*(h*_*c*_*)* the projected contact area under load [mm^2^], $v$_s_ Poisson’s ratio of the specimen with $v$_s_ = 0.4 and $v$_i_ the Poisson’s ratio of the indenter with $v$_I_ = 0.3, and S the contact stiffness evaluated from the force removal curve [24, 25].

**Fig. 3:**
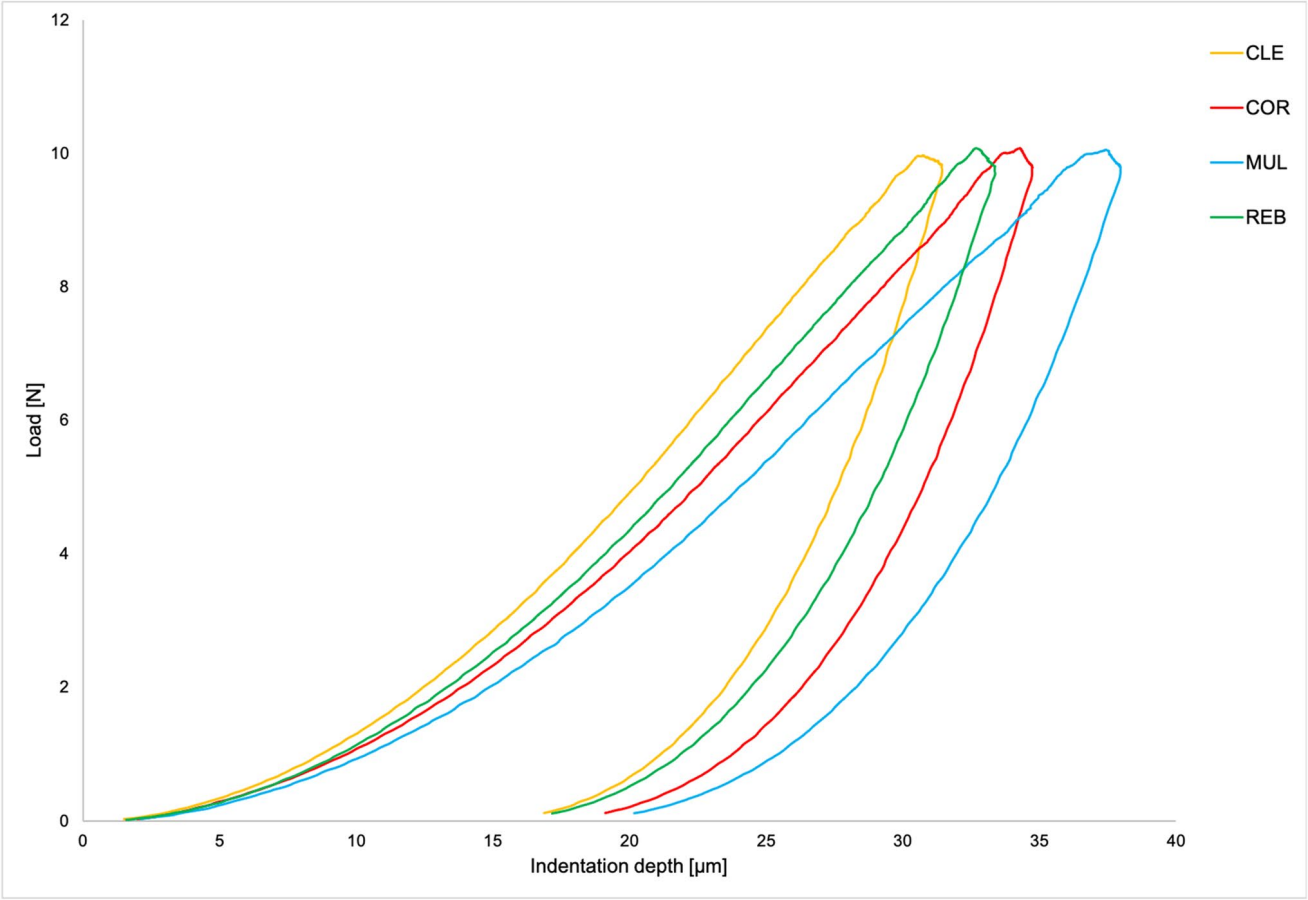
Load-indentation depth curves for one light-polymerized specimen of each core build-up material

Exemplarily load-indentation depth curves for one light-polymerized specimen of each core build-up material are depicted in Fig. 3.

### Measurement of the biaxial flexural strength (BFS)

**Fig. 4:**
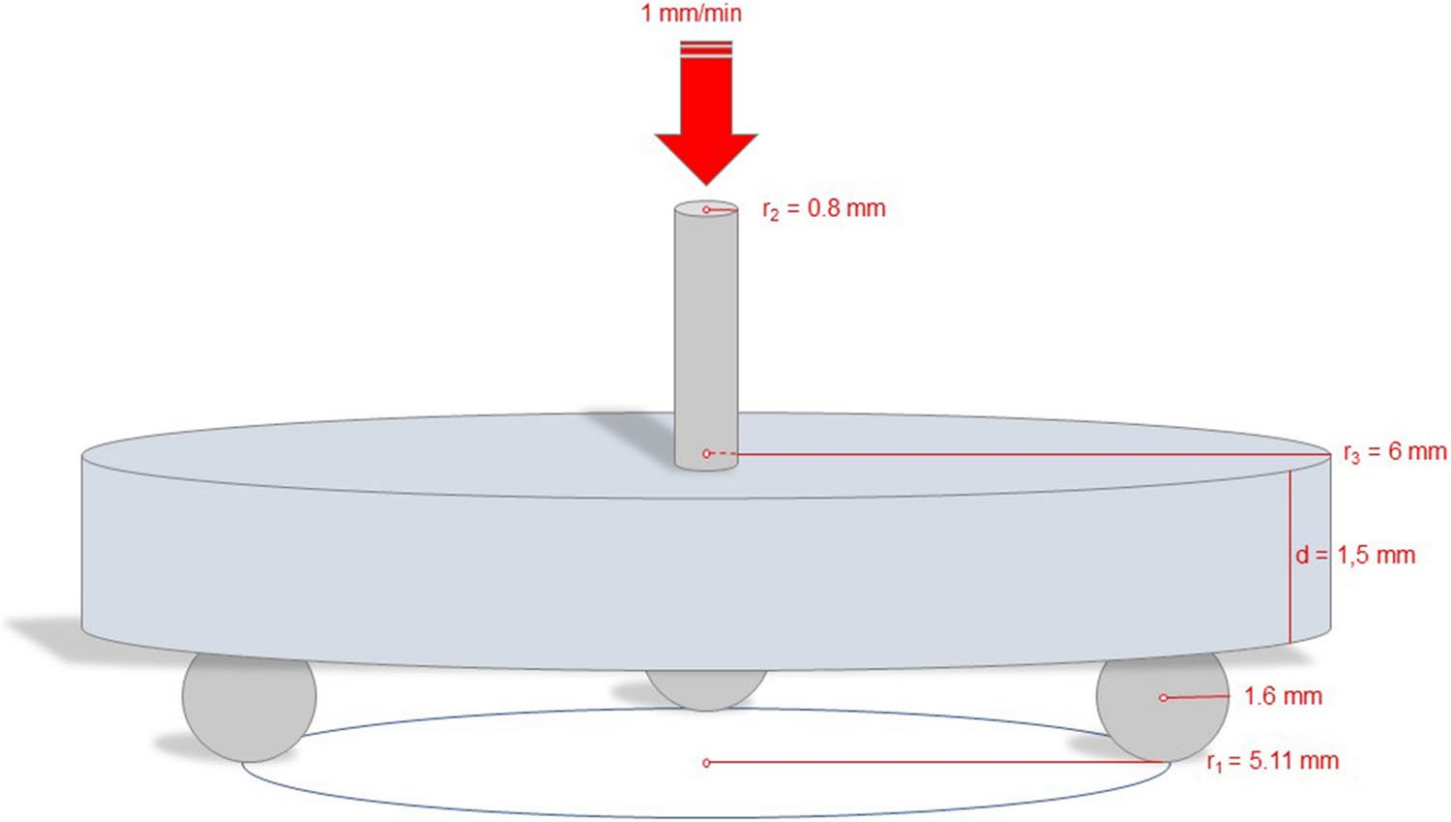
Schematic presentation of the biaxial flexural strength test set-up

Biaxial flexural strength measurements were performed at a room temperature of 23 °C using the universal testing machine Z010 (ZwickRoell). The test was performed according to DIN EN ISO 6872:2008 [26]. The thickness of each specimen was determined using a digital micrometer screw (IP65, Mitutoyo, Kawasaki, Japan) with an accuracy of ± 4 µm before specimens were placed on a jig with three balls. The tempered steel balls had a diameter of 3.2 mm and formed an equilateral triangle. Loading with a crosshead speed of 1 mm/min was applied with a 1.6-mm diameter plunger in the center of each specimen until failure occurred (Fig. 4).

BFS was calculated with the following formula:

$$\sigma = {-0.2387P}^{*}(X-Y)/{d}^{2}$$

where *P* is fracture load [N], *d* is specimen thickness [mm], and the coefficients *X* and *Y* represent:

$$X=(1+\upsilon ) ln {\left[\left(\frac{{r}_{2}}{{r}_{3}}\right)\right]}^{2}+[(1-\upsilon )/2]{({r}_{2}/{r}_{3})}^{2}$$

$$Y=(1+\upsilon ) [1+ln [{\left(\frac{{r}_{1}}{{r}_{3}}\right)}^{2}]+(1-\upsilon ) {({r}_{1}/{r}_{3})}^{2}$$

where *υ* is the Poisson’s ratio (*υ* = 0.4), *r*_1_ is the radius of the support circle formed by the three tempered steel balls [mm], *r*_2_ is the radius of the loaded area [mm], and *r*_3_ is the specimen radius [mm].

### Statistical analysis

Descriptive analysis was calculated, and deviations from the normal distribution were tested using the Kolmogorov–Smirnov test. For non-parametric analysis, Mann–Whitney *U* and Kruskal–Wallis tests were performed. Correlation coefficients were determined using Spearman’s correlation. The Weibull modulus was calculated with the maximum likelihood estimation method and 95% confidence interval [27]. For all statistical analyses, a *p*-value below 0.05 was interpreted as statistically significant (IBM Statistics SPSS 26.0, IBM, Armonk, USA).

## Results

The results of the descriptive analyses are presented in Tables 2 and 3. With the Kolmogorov–Smirnov test showing a violation of the assumption of normality for 12.5% of the tested groups, non-parametric tests were performed.

**Table 2:** Descriptive statistics (min/median/max) for the degree of conversion [%], Martens hardness [N/mm2], and elastic indentation modulus [kN/mm2] for all tested groups

	CLE	COR	MUL	REB
i) Light-polymerization				
DC	54.2/61.5/67.8^b,A^	62.0/67.1/74.2^b,B^	63.0/67.7/76.0^b,B^	49.0/72.1/76.0^b,B^
HM	286/304/400*^b,B^	218/323/378^b,B^	188/254/319^b,A^	193/336/387^b,B^
E_IT_	6.83/7.88/11.1^b,B^	5.23/8.32/11.0^b,B^	4.77/6.43/9.23^a,A^	4.10/8.38/10.3^b,B^
ii) Self-polymerization				
DC	29.3/44.6/48.9*^a,A^	49.7/55.7/60.5^a,B^	44.7/52.6/59.4^a,B^	34.1/50.6/63.0^a,AB^
HM	127/165/224^a,A^	197/251/310^a,C^	128/215/254*^a,B^	140/230/304^a,BC^
E_IT_	3.20/4.15/6.43^a,A^	4.53/6.52/8.60^a,B^	3.07/5.82/7.73^a,B^	3.40/5.90/8.37^a,B^

^*^Not normally distributed

abc Different letters present significant differences between polymerization modes within one core build-up material

ABC Different letters present significant differences between core build-up materials within one polymerization mode

**Table 3:** Descriptive statistics (min/median/max) for the biaxial flexural strength [MPa] and Weibull moduli (median [95% CI]) for all tested groups

	CLE		COR		MUL		REB	
i) Light-polymerization								
BFS (min/median/max)	82.4/130/168^b,A^		134/164/180*^b,B^		101/144/167^a,A^		94.0/144/184^a,AB^	
Weibull modulus (median [95% CI])	5.5^a,A^	[2.9; 10.0]	13.2^b,B^	[7.1; 23.9]	6.8^a,A^	[3.6; 12.4]	5.5^a,A^	[2.9; 10.0]
ii) Self-polymerization								
BFS (min/median/max)	66.3/89.3/101^a,A^		94.0/134/154^a,B^		104/124/138^a,B^		77.6/127/162^a,B^	
Weibull modulus (median [95% CI])	9.2^a,AB^	[5.0; 16.8]	6.9^a,A^	[3.7; 12.6]	13.3^b,B^	[7.2; 24.1]	5.4^a,A^	[2.8; 9.8]

^*^Not normally distributed

abc Different letters present significant differences between polymerization modes within one core build-up material

ABC Different letters present significant differences between core build-up materials within one polymerization mode

**Fig. 5:**
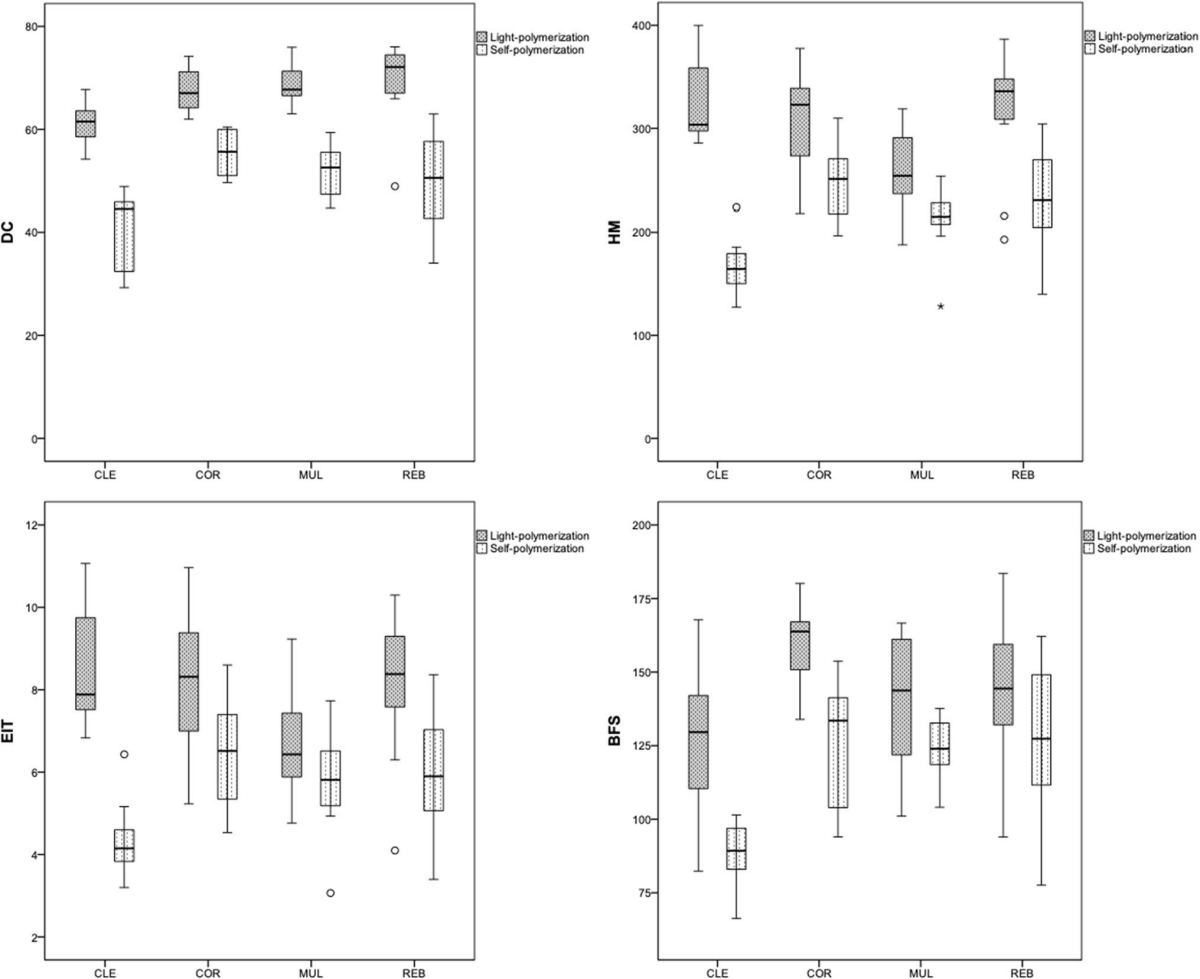
Box plots for the tested chemical and mechanical properties of the four core build-up materials as a function of the polymerization mode

For the DC (*p* < 0.001) and the HM (*p* < 0.001–0.009), light-polymerization led to higher values than self-polymerization for all four tested core build-up materials. For the E_IT_, higher values were also observed after light-polymerization for all groups (*p* < 0.001–0.009) except for MUL, where no difference was observed between the two polymerization modes (*p* = 0.119). The polymerization mode furthermore showed an influence on the BFS of CLE and COR (*p* = 0.001), where light-polymerization resulted in higher values, but did not impact the BFS of MUL or REB (*p* = 0.094–0.326; Fig. 5). For COR, light-polymerization resulted in a higher Weibull modulus than observed after self-polymerization. The opposite trend was observed for MUL, where self-polymerization led to a higher Weibull modulus. The polymerization mode did not influence the Weibull moduli of CLE or REB.

After light-polymerization, CLE showed a lower DC than all other groups (*p* < 0.001). MUL presented a lower HM and E_IT_ than the three other materials (*p* = 0.004–0.014). Regarding the BFS, CLE and MUL presented the lowest values, while the highest BFS was observed for COR (*p* = 0.020). COR furthermore presented a higher Weibull modulus than the other tested core build-up materials.

After self-polymerization, CLE presented a lower DC than MUL and COR (*p* < 0.001). For the HM, the lowest values were reported for CLE, while REB and COR led to the highest values (*p* < 0.001). For the E_IT_ (*p* = 0.002) and BFS (*p* < 0.001), CLE showed lower values than all other groups. REB and COR showed lower Weibull moduli than observed for MUL.

Positive correlations between all four tested parameters (*R* = 0.527–0.963, *p* < 0.001) were found.

## Discussion

The aim of this investigation was to examine the chemical and mechanical properties of four different core build-up materials after light- and self-polymerization. The tested null hypotheses, that neither the polymerization mode nor the material presented an influence on the DC, HM, E_IT_, or BFS, had to be rejected.

When regarding the impact of the polymerization mode on the tested properties, it could be shown that light-polymerization led to higher DC and HM values than observed after self-polymerization for all core build-up materials. In Fig. 2, the different proportion of the two peaks at 1640 cm^−1^, representing the residual unpolymerized methacrylate C = C stretching mode, and at 1610 cm^−1^, showing the aromatic C = C stretching mode used as an internal standard before and after polymerization, after light-polymerization (center) or self-polymerization (right) is clearly visible. Consistently, light-polymerization resulted in higher E_IT_ and BFS values for the majority of the tested groups except for the two core build-up materials MUL and REB that showed comparable E_IT_ values (MUL) and BFS results (MUL and REB) for both polymerization modes. Light-polymerization furthermore resulted in a higher reliability on the part of COR. As the kinetics of chain propagation and termination proceed similarly in light- and self-initiated polymerization reactions, differences have to be sought in the effectiveness of the initiation reaction, induced by photoactivation or redox reaction. Previous investigations have reported the additional effect of light irradiation to be material dependent, reporting a wide range of outcomes from no impact to a high impact on the resulting material properties [10, 17, 18, 20, 28]. In line with the present findings, the sole use of self-polymerization has been observed to lead to a reduced DC and lower mechanical properties [10, 17, 18, 29]. However, with post-polymerization continuing over a period of up to 24 h after the start of the reaction, the DC and the interrelated mechanical properties of self-polymerized specimens may increase over time and align with those reported for their light-polymerized counterparts [30, 31]. A previous investigation on the polymerization kinetics of bulk-fill resin composites reported self- and light-polymerization to only compete in the initial stage of polymerization. Just 11 min after the initiation reaction, the DC vs. time curves were perfectly superposed in all analyzed groups [30]. The differences observed between self- and light-polymerization in the present investigation may hence subside over time. If this were the case, the advantage of light-polymerization may be primarily seen in the improved clinical applicability. In this context, the approved thickness of the individual increments also plays a crucial role. While the manufacturers indicate CLE, COR, and REB to only be applied in increments of 2–3-mm thickness, the operating instructions of MUL do not include such directives. With previous investigations reporting a reduced DC and hardness at an increased distance from the surface [32, 33], future investigations should examine whether the specified increment thicknesses of the tested core build-up materials need to be adjusted to ensure the best possible clinical outcome.

The choice of material also presented an impact on the tested chemical and mechanical parameters. After light-polymerization, CLE showed the lowest DC and low BFS values when compared with the other tested core build-up materials. While the employed light-polymerization duration should be considered a plausible cause for this observation [32, 34, 35], with CLE and MUL (the core build-up material that presented the lowest Martens parameters after light-polymerization) both being cured for a shorter duration of only 10 s as compared to 20 s for COR and 40 s for REB, the same trend was applied after self-polymerization, where CLE presented low DC values and the lowest HM, E_IT_, and BFS results. With CLE specimens being manufactured adhering to a self-polymerization duration of 6 min (as specified by the manufacturer), a period that exceeds those of the other tested materials, the reason for CLE’s performance may originate from the resin composite’s composition. When regarding the limited information provided by the manufacturers, CLE can be differentiated from the other three materials by the apparent absence of the urethane dimethacrylate (UDMA) monomer. With UDMA possessing a lower viscosity and thus higher mobility of the monomers than bisphenol A-glycidyl methacrylate (Bis-GMA), the DC and the mechanical properties of resin composites increase [36]. The manufacturer furthermore indicates a variation of fillers (e.g., silanated barium glass and aluminum oxide), the photoinitiator dl-Camphorquinone, additional unspecified initiators, accelerators, and pigments in the composition of MUL. While all these constituents influence the initiation, chain propagation, and ultimate termination of the polymerization reaction and hence the chemical and mechanical properties of the resin composites, a further comparison between materials based on the very limited information provided by the other three manufacturers would be unsubstantiated.

After light-polymerization, the core build-up material MUL presented the lowest HM and E_IT_ (as seen in Fig. 3). As this differentiation did not persist after self-polymerization, with MUL showing superior values to CLE (HM and E_IT_) that were in the same range as observed for COR (E_IT_) and REB (HM and E_IT_), it may be assumed that in comparison with the other materials, MUL’s light-induced initiation of the radical polymerization reaction was impaired. This theory is underlined by MUL being the only material where self-polymerization resulted in a higher Weibull modulus than observed after light-polymerization. Likely causes for this observation include an insufficiently long light exposure for 10 s [32, 34, 35] or an imbalance in the composition of MUL. While dibenzoyl peroxide is indicated as the employed chemical initiator, the included photoinitiators are not specified. An inadequate amount, uneven distribution, or mismatch of the components included in the light-induced start reaction (e.g., a lack of co-initiators) could explain MUL’s high inhomogeneity after light-polymerization that is quantified by its low Weibull modulus. While not ideally aligned for light-polymerization, the composition of MUL might, however, allow for an optimal self-polymerization reaction. This theory is highlighted by MUL showing a higher reliability than COR and REB after self-polymerization.

COR, on the other hand, showed a high DC and HM after self-polymerization and the highest BFS after light-polymerization when compared with the other tested core build-up materials. After light-polymerization, COR furthermore presented the highest Weibull modulus. With the processing guidelines of COR indicating a light exposure of 20 s, that lies between that employed for CLE/MUL (10 s) and REB (40 s), and the duration of self-polymerization being set at 2–3 min, which is lower than that specified for its competitors (4–6 min), the reasons for COR’s excellent chemical and mechanical performance seem to stem from its composition. In this context, its component propylidynetrimethyl trimethacrylate (TMPTMA) could play a key role, as this monomer is characterized by its ability to speed up the polymerization by reacting with an amine [37]. The high reliability observed for COR after light-polymerization did, however, not transfer to the material’s properties after self-polymerization, where in combination with REB, COR showed a lower Weibull modulus than observed for MUL.

Considering the present findings, the use of light-polymerization in conjunction with the resin composite COR may thus be recommended to increase the chemical and mechanical properties of core build-ups. This strategy allows for increments to be placed in 3 mm thicknesses and includes a reasonable light exposure period of 20 s per increment. As the Spearman's *ρ* showed a positive correlation between the four parameters DC, HM, E_IT_, and BFS, the chemical and mechanical properties of the four tested core build-up materials seem to be linked. Future investigations may thus focus on the mechanical properties of this material group, as these are more readily obtained, and transfer gained insights to their chemical performance. A comparison of the E_IT_ values reported for the four tested materials (median: 4.15–8.38 kn/Nmm^2^) to that of dentin (mean: 19.89 kn/Nmm^2^) [38] and enamel (median: 51.1 kn/Nmm^2^ [22]; mean: 80.35 kn/Nmm^2^) [38] underlines the role resin composite core-build up materials play in imitating the natural tooth in conjunction with a ceramic restoration (median: 49.7 kn/Nmm^2^) [22].

The findings of the present investigation have to be evaluated in regard to their limitations. These encompass the in vitro study design and the limited number of examined core build-up materials. Although the employed methods yielded results with a low standard deviation, underlining the reliability of the measurement set-ups, a variation in BFS values dependent of the performing laboratory has been reported in a round robin test [39]. A comparison to other investigations using different workflows, set-ups, or measurement methods may thus be difficult [39]. Uniform polymerization durations, both for light- and self-polymerization, could enhance our understanding of differences observed between materials based solely on their composition. Interestingly, a previous investigation testing a wide range of different core-build up resin composites that employed uniform polymerization durations did not observe differences between the four materials examined in the present investigation in regard to their flexural strength and flexural modulus [10]. The therein reported strength values were slightly lower than those observed in the present investigation, as is to be expected due to the differing test set-up (three-point vs. biaxial flexural strength measurement). Longitudinal measurements are warranted to confirm or revoke the presently observed differences between the two polymerization modes in the course of time. As material properties differ throughout a light-polymerized specimen depending on the distance to the surface [32, 40], with the attenuation of light following an exponential decay [41], future investigations should furthermore investigate whether the observed trends can also be observed at different distances from the surface and for varying increment thicknesses. In the present investigation, the shade of the tested materials varied. For CLE, MUL, and REB, a white shade was chosen, while COR, which is only available as tooth-colored, was examined in a darker shade. With the material color arising from doping with pigments that can influence the amount of light passing through the resin composite due to their opaque character, future investigations should investigate whether the observed differences between the tested materials persist for different shade options [9, 16].

## Conclusions

Based on the findings of this investigation, the following conclusions can be drawn:

1. For the tested resin composite core build-up materials, light-polymerization led to similar or superior values for the degree of conversion, Martens hardness, elastic indentation modulus, and biaxial flexural strength than observed after self-polymerization.
2. Among the tested materials, COR should represent the resin composite core build-up material of choice due to its high chemical and mechanical properties and its high reliability after light-polymerization.
3. The examined chemical (degree of conversion) and mechanical (Martens hardness, elastic indentation modulus, and biaxial flexural strength) properties showed a positive correlation.
